# Pomolic acid induces ferroptosis-mediated cell death in non-small cell lung cancer

**DOI:** 10.3389/fphar.2025.1567942

**Published:** 2025-06-04

**Authors:** Wenbin Ji, Yanbin Zhang, Wenhao Ji, Hui Zhang, Biao Qin, Xiao-Liang Xing, Zaiqi Zhang

**Affiliations:** ^1^ The First Clinical Medical College, Ningxia Medical University, Yinchuan, Ningxia, China; ^2^ School of Medicine and School of Public Health and Emergency Management, Hunan University of Medicine, Huaihua, Hunan, China; ^3^ School of Medical Information Engineering, Gannan Medical University, Ganzhou, Jiangxi, China

**Keywords:** PA, ferroptosis, NSCLC, network pharmacology, ferroptosis-related proteins

## Abstract

**Introduction:**

Pomolic acid (PA), a bioactive compound derived from Potentilla freyniana Bornm., is used palliatively for non-small cell lung cancer (NSCLC) in China’s Dongzu region, with some reports of clinical efficacy. However, the specific underlying molecular mechanisms remain unclear. This study aimed to identify the core targets of PA and explore its function and potential mechanisms in NSCLC.

**Methods:**

Network pharmacological analysis was utilized to identify the core targets of PA. In vitro functional studies were performed using NSCLC cells to investigate PA’s effects on cell death and proliferation. Assays were conducted to measure hallmarks of ferroptosis, including glutathione (GSH) depletion, iron (Fe^2+^)-dependent lipid peroxidation, and elevated reactive oxygen species (ROS) levels. Protein expression levels of key anti-ferroptotic factors (SLC40A1, SLC7A11, GPX4) and pro-ferroptotic proteins (ACSL4, HO-1) were assessed. Quantitative PCR (qPCR) was used to determine mRNA expression levels of genes negatively regulating ferroptosis (GPX4, SLC7A11, NRF2).

**Results:**

PA effectively induced cell death and inhibited proliferation in NSCLC cells. Characteristic hallmarks of ferroptosis were observed, including GSH depletion, Fe^2+^-dependent lipid peroxidation, and increased ROS levels. Protein expression levels of SLC40A1, SLC7A11, and GPX4 were significantly downregulated, while ACSL4 and HO-1 were markedly upregulated. mRNA expression levels of GPX4, SLC7A11, and NRF2 were also significantly reduced.

**Discussion:**

These findings suggest that PA exerts its anticancer effects primarily through ferroptosis induction. The observed modulation of key ferroptosis-related proteins and genes supports this mechanism. Therefore, PA may serve as a promising therapeutic agent for NSCLC treatment.

## 1 Introduction

Lung cancer represents a primary cause of global cancer-related mortality, responsible for approximately 631,000 deaths annually (Siegel et al., 2021). Contrary to trends in Western nations, lung cancer incidence continues to increase in China (Chen et al., 2022). NSCLC is the predominant subtype, accounting for 80%–85% of lung cancer cases (Hendriks et al., 2024). Standard NSCLC therapeutic modalities encompass surgery, chemotherapy, radiotherapy, targeted therapy, and immunotherapy. Surgical resection is considered appropriate for about 50% of patients with stage I and II NSCLC (Howington et al., 2013). In contrast, only a small proportion of stage III NSCLC patients are surgical candidates; most patients in this category receive conventional treatments, primarily chemotherapy or radiotherapy (Ramnath et al., 2013). Adjuvant chemoradiotherapy is frequently administered to patients following surgical intervention. However, these treatments often cause severe adverse effects, significantly compromising the patient’s quality of life. Despite therapeutic progress, NSCLC mortality remains high (Howlader et al., 2020). Therefore, an urgent need exists for the development of novel therapeutic strategies and effective anti-NSCLC agents.

The theory and practice of Traditional Chinese Medicine (TCM) in cancer treatment are well-established, providing a solid foundation for contemporary applications in oncology. For example, the Zishen Tongyang Huoxue decoction is reported to regulate mitochondrial membrane permeability via VDAC1, which affects Mitochondrial Quality Control (MQC) through β-tubulin and inhibits mitochondrial apoptosis (Chang et al., 2024b; Chang et al., 2024c). In the Dong ethnic region of China, *Potentilla freyniana* Bornm. (Madeng’ai). It is used for the palliative treatment of NSCLC, with occasional reports of clinical efficacy. PA is a bioactive constituent isolated from this plant. Mounting evidence indicates that PA possesses significant anti-cancer activity in various tumor models (Salvador et al., 2012; Pereira et al., 2018; Liu et al., 2023). Specifically, PA demonstrates anti-acute myeloid leukemia effects by inducing cell death, inhibiting cell proliferation, and suppressing topoisomerase activity (Pereira et al., 2018). PA also exerts anti-colon cancer activity through the induction of autophagy and the promotion of apoptosis (Liu et al., 2023). However, the precise molecular mechanisms underlying the effects of PA in NSCLC remain to be fully elucidated.

Ferroptosis, a distinct form of regulated cell death, was first described in 2012 (Dixon et al., 2012). The hallmark of ferroptosis is iron-dependent lipid peroxidation, a process leading to the accumulation of lipid hydroperoxides and dysregulation of iron metabolism, ultimately generating lethal levels of reactive oxygen species (ROS) (Dixon et al., 2012). Modulation of ferroptosis is considered a promising strategy to overcome resistance to conventional cancer therapies, particularly in the context of tumor recurrence and drug resistance (Lei et al., 2022; Wang et al., 2023). Consequently, ferroptosis attracts significant research attention as a novel mechanism for inducing tumor cell death. PA exhibits broad pharmacological activities, including anti-inflammatory, antioxidant, and anti-cancer effects (Ghante and Jamkhande, 2019). This study, therefore, investigates the potential role of ferroptosis modulation in the anti-NSCLC activity of PA.

## 2 Materials and methods

### 2.1 Cell culture

Human normal lung epithelial cells (BEAS-2B) and human NSCLC cells (H1299 and A549) were obtained from the American Type Culture Collection. BEAS-2B cells were cultured in Dulbecco’s Modified Eagle Medium (DMEM) supplemented with 10% fetal bovine serum (FBS) and 1% Penicillin-Streptomycin (PS). H1299 and A549 cells were cultured in RPMI-1640 medium supplemented with 10% FBS and 1% Penicillin-Streptomycin (PS). All cells were maintained in a 37°C, 5% CO_2_ incubator.

### 2.2 Reagents and antibodies

PA was purchased from Shanghai Aladdin Biochemical Technology Co., Ltd. and prepared as a stock solution of 10 mM dimethyl sulfoxide (DMSO), then aliquoted and stored at −80°C. Deferoxamine (HY-B0988), Ferrostatin-1 (HY-100579), necrostatin-1 (HY-15760), Z-VAD-FMK (HY-16658B), chloroquine (HY-17589A), erastin (HY-15763), and liproxstatin-1 (HY-12726) were purchased from MedChemExpress (MCE).

The antibodies used in the present study were anti-ACTIN (Abcam, ab179467), anti-GPX4 (MCE, HY-P80450), anti-FTH1 (MCE, HY-P80670), anti-xCT (MCE, HY-P80935), anti-Heme Oxygenase-1 (HO-1) (Abcam, ab13248), anti-SLC40A1 (Abcam, ab239583), anti-ACSL4 (Biogot, BS71431), anti-NRF2 (Cell Signaling, 12721), goat anti-rabbit IgG H&L (HRP) (Abcam, ab6721), and rabbit anti-mouse IgG H&L (HRP) (Abcam, ab6728). All antibodies were used at a 1:1000 dilution.

### 2.3 Candidate gene identification

The SMILES number for “Pomolic acid” was retrieved from the PubChem database (https://pubchem.ncbi.nlm.nih.gov/) using it as a keyword. Drug target prediction was performed using the SwissTargetPrediction database (http://www.swisstargetprediction.ch/), SuperPreD database (https://prediction.charite.de/), and TargetNet database (http://targetnet.scbdd.com/), with species set to *Homo sapiens*.

The OMIM (http://www.omim.org/), GeneCards (https://www.genecards.org/), and DisGeNET (https://disgenet.com/) databases were used to retrieve targets related to NSCLC by entering the keyword “non-small-cell lung cancer.” The top 5,000 targets were selected from the GeneCards database and further verified and converted using the UniProt database (https://www.uniprot.org/).

The FerrDb database (http://www.zhounan.org/ferrdb) was used to identify marker genes, driver genes, and inhibitory genes related to ferroptosis, selecting only “Human” genes for the query.

The results were exported into Excel tables for further cleaning and removing duplicate genes. Venn analysis was employed to perform intersection analysis on the drug, disease, and ferroptosis-related targets, identifying the common targets.

### 2.4 Network pharmacology analysis

The intersection targets were imported into the STRING database (https://string-db.org/) to construct a protein-protein interaction (PPI) network, with species limited to *Homo sapiens* and a confidence score threshold set at 0.4. The PPI network was visualized using Cytoscape v3.10.0, and core targets were identified based on the degree value. Node size and color depth in the network diagram reflected the degree of connectivity of each target.

Gene Ontology (GO) functional enrichment and Kyoto Encyclopedia of Genes and Genomes (KEGG) pathway enrichment analyses were performed on the intersection targets using the DAVID 6.8 database with the default parameter (https://davidbioinformatics.nih.gov/) (Kanehisa and Goto, 2000; Kanehisa, 2019; Kanehisa et al., 2023). GO analysis was conducted using a P-value threshold, selecting the top 10 biological processes with the smallest P-values, which were visualized in a bar graph. For KEGG analysis, the top 20 most significantly enriched signaling pathways were selected using the same P-value screening condition, and the results were visualized in a bubble chart.

### 2.5 Cell viability assay

The effect of PA on NSCLC cell viability is determined using the Cell Counting Kit-8 (CCK-8) assay (LJ621; Dojindo). Cells are seeded into 96-well plates at a density of 1 × 104 cells/well. After overnight adherence, cells are treated with various concentrations of PA or vehicle control for 24 h. Subsequently, CCK-8 reagent (10 µL/well) is added, and plates are incubated for 1 h at 37°C in a 5% CO_2_ atmosphere. Absorbance is measured at 450 nm using a microplate reader. Cell viability is expressed as a percentage relative to vehicle-treated control cells.

### 2.6 Colony formation assay

The long-term effect of PA on cell proliferation is assessed via the colony formation assay. H1299 and A549 cells are seeded sparsely in 6-well plates (e.g., 500 cells/well). Cells are cultured in a medium containing specified concentrations of PA or vehicle control for 24 h. The medium is refreshed every 3 days. After 10 days of incubation, visible colonies are fixed (e.g., with 4% paraformaldehyde or ice-cold methanol) and stained with 0.5% Crystal Violet solution (G1064; Solarbio) for 30 min. Plates are rinsed, air-dried, and imaged. Colonies containing ≥50 cells are counted manually under blinded conditions or automatically using ImageJ software.

### 2.7 Cell cycle and apoptosis

Analysis by Flow Cytometry Apoptosis induction by PA in NSCLC cells is evaluated using the Annexin V-APC/PI Apoptosis Detection Kit (A217; Elabscience). Cell cycle distribution analysis is performed using the Cell Cycle Assay Kit (A352; Elabscience). Briefly, cells are treated with PA (specified concentrations and duration) or vehicle control for 24 h. For apoptosis analysis, cells are harvested, washed, resuspended in binding buffer, and stained with Annexin V-APC and Propidium Iodide (PI) according to the kit protocol. For cell cycle analysis, harvested cells are fixed and stained with PI containing RNase A. Stained cells are analyzed using a flow cytometer. Data acquisition and analysis are performed using CytExpert software.

### 2.8 QRT-PCR

Total RNA and protein were extracted using an RNA/Protein Extraction Kit (Beyotime, R0018M). The RNA was then reverse transcribed into cDNA using the All-in-one First Strand cDNA Synthesis Kit Ⅲ (with dsDNase) (Seven, SM135). The mRNA expression was analyzed using 2× SYBR Green qPCR MasterMixⅡ (Seven, Sm143). The qPCR primer sequences used in this study are listed in Table 1. The qRT-PCR results were analyzed using the 2^−ΔΔCT^ method.

**TABLE 1:** Primers for qRT-PCR.

Gene	Forward primer	Reverse primer
*SLC7A11*	TCC​TGC​TTT​GGC​TCC​ATG​AAC​G	AGA​GGA​GTG​TGC​TTG​CGG​ACA​T
*GPX4*	ACA​AGA​ACG​GCT​GCG​TGG​TGA​A	GCC​ACA​CAC​TTG​TGG​AGC​TAG​A
*NRF2*	CAC​ATC​CAG​TCA​GAA​ACC​AGT​GG	GGA​ATG​TCT​GCG​CCA​AAA​GCT​G
*ACTIN*	CAC​CAT​TGG​CAA​TGA​GCG​GTT​C	AGG​TCT​TTG​CGG​ATG​TCC​ACG​T

### 2.9 Determination of Intracellular Fe^2+^ levels

Intracellular Fe^2+^ levels were determined using FerroOrange (Dojindo, F374). Cells were treated with or without PA for 24 h, then incubated with a FerroOrange working solution (1 μmol/L) for 30 min at 37°C in a 5% CO_2_ incubator. The fluorescence signal was observed and photographed under a confocal microscope.

### 2.10 Measurement of ROS

The intracellular ROS assay was performed using the fluorescent probe DCFH-DA (Beyotime, S0033). A549 and H1299 cells were treated with or without PA for 24 h. After treatment, cells were harvested, suspended in DCFH-DA working solution, and incubated at 37°C for 30 min. The fluorescence signal was then detected by flow cytometry.

### 2.11 Malondialdehyde (MDA) assay

Lipid peroxidation is evaluated by quantifying MDA, a major end-product, using the Lipid Peroxidation MDA Assay Kit (S0131; Beyotime). Following experimental treatments, cells are harvested, washed, and lysed according to the kit manufacturer’s protocol. The absorbance of this adduct is measured spectrophotometrically at 532 nm using a microplate reader. MDA concentration is calculated based on a standard curve generated using MDA standards provided in the kit and normalized to total protein content.

### 2.12 GSH assay

Intracellular levels of reduced GSH and oxidized glutathione (GSSG) are determined using the GSH and GSSG Assay Kit (S0053; Beyotime), which employs a kinetic enzymatic recycling method. Cells are treated, harvested, and processed following the kit’s instructions to separate GSH and GSSG fractions or measure total glutathione. Absorbance changes are monitored kinetically at 412 nm using a microplate reader. Concentrations of total glutathione and GSSG are calculated from standard curves, and the GSH concentration is determined by subtracting GSSG from total glutathione.

### 2.13 Western blotting

Cells treated with PA or vehicle control are harvested and lysed on ice using RIPA Lysis Buffer (P0013B; Beyotime Biotechnology) supplemented with a protease and phosphatase inhibitor cocktail. Total protein concentration in the lysates is quantified using the BCA Protein Assay Kit (P0010; Beyotime Biotechnology). Equal amounts of protein (typically 20–40 µg) are resolved by SDS-PAGE (using 8%–15% acrylamide gels, depending on target protein size) and transferred onto PVDF membranes. Membranes are blocked for 1 h at room temperature with 5% non-fat dry milk or 5% BSA in TBST (Tris-buffered saline, 0.1% Tween-20). Membranes are then incubated overnight at 4°C with specific primary antibodies (listed in Section 2.2) diluted in blocking buffer according to manufacturer recommendations. After washing steps in TBST, membranes are incubated with appropriate HRP-conjugated secondary antibodies (listed in Section 2.2) diluted in a blocking buffer for 1 h at room temperature. Protein bands are detected using an Enhanced Chemiluminescence (ECL) substrate kit (P0018S; Beyotime) and visualized with a chemiluminescence imaging system. Relative band densities are quantified using densitometry software (e.g., ImageJ v1.53, NIH or Image Lab Software, Bio-Rad) and normalized to a loading control (e.g., ACTIN).

### 2.14 Statistical analysis

All quantitative experiments are performed independently at least three times. Data are presented as mean ± standard deviation (SD). Statistical significance is evaluated using GraphPad Prism software or SPSS Statistics. Normality and homogeneity of variances are assessed prior to parametric testing. Comparisons between two experimental groups are made using an unpaired, two-tailed Student’s *t*-test. For comparisons involving three or more groups, one-way analysis of variance (ANOVA) is employed, followed by Tukey’s or Dunnett’s *post hoc* test for multiple comparisons, as appropriate. A P-value <0.05 is considered statistically significant.

## 3 Results

### 3.1 PA induces cell death and inhibits proliferation in NSCLC cells

**FIGURE 1:**
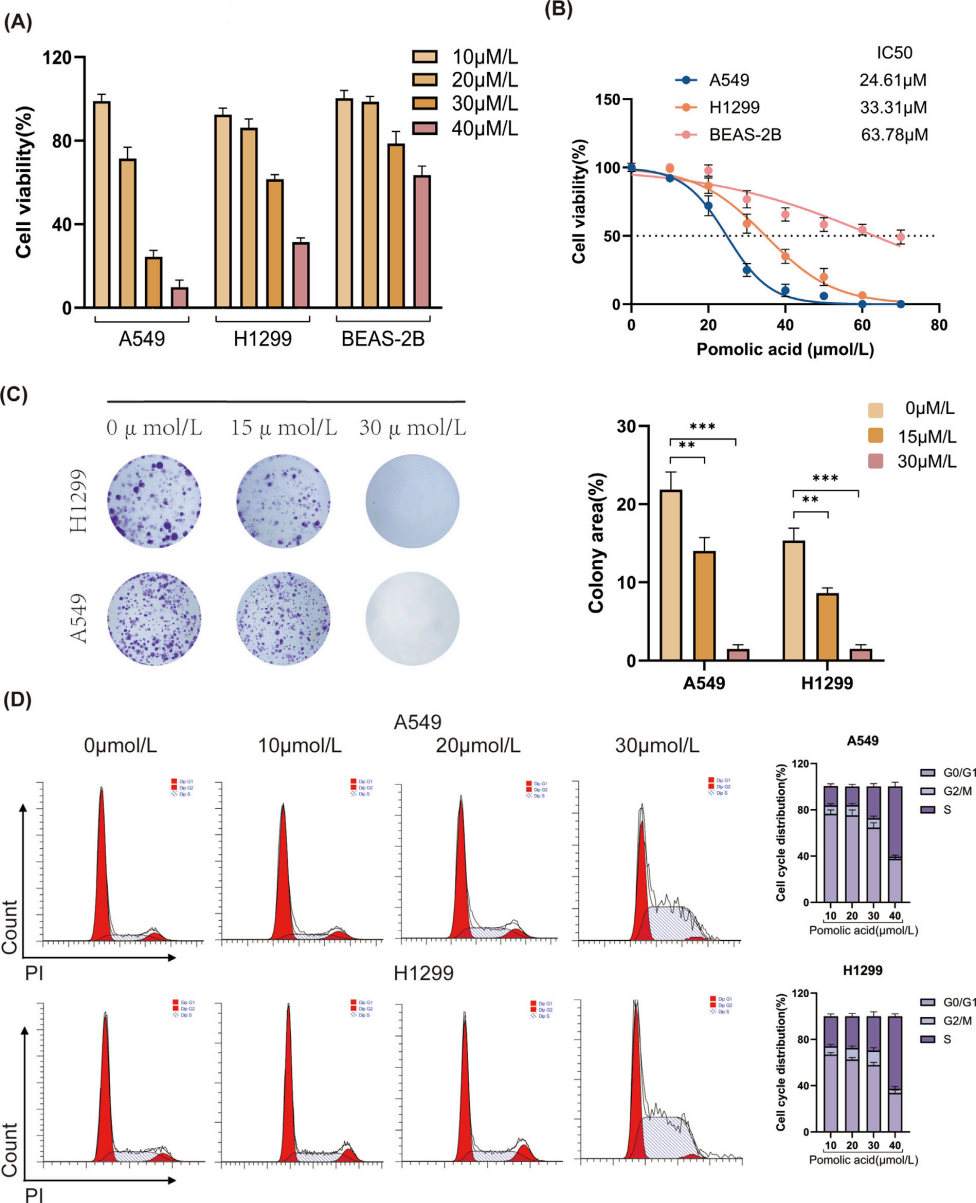
Effects of PA on cell viability and colony formation in A549 and H1299 cells. **(A,B)** A549, H1299, and BEAS-2B cells were treated with different concentrations of PA for 24 h. Cell viability was then detected using CCK-8, and IC_50_ values were calculated. **(C)** Effects of PA on colony formation of A549 and H1299 cells and quantitative analysis of colony counts. **(D)** The impact of PA on the cell cycle of A549 and H1299 cells was analyzed by flow cytometry, followed by statistical analysis of the results. *P < 0.05, **P < 0.01, ***P < 0.001.

To examine the anti-cancer potential of PA against NSCLC, the viability of normal human lung epithelial cells (BEAS-2B) and NSCLC cell lines (H1299, A549) is assessed following treatment with various concentrations of PA for 24 h. The results demonstrate that PA exerts dose-dependent cytotoxicity against A549 and H1299 cells while exhibiting minimal toxicity toward the non-cancerous BEAS-2B cells within the tested concentration range (Figures 1A,B).

Next, the effect of PA on the long-term proliferative capacity of NSCLC cells is evaluated using colony formation assays. These experiments reveal that PA significantly suppresses the colony-forming ability of both H1299 and A549 cells in a dose-dependent manner (Figure 1C). To further investigate the mechanism underlying the inhibition of proliferation, cell cycle analysis is performed using flow cytometry. In both A549 and H1299 cells, PA treatment results in cell cycle arrest, evidenced by a dose-dependent increase in the percentage of cells residing in the S and G2/M phases compared to vehicle-treated control cells (Figure 1D).

### 3.2 Network pharmacology analysis links PA to ferroptosis in NSCLC

**FIGURE 2:**
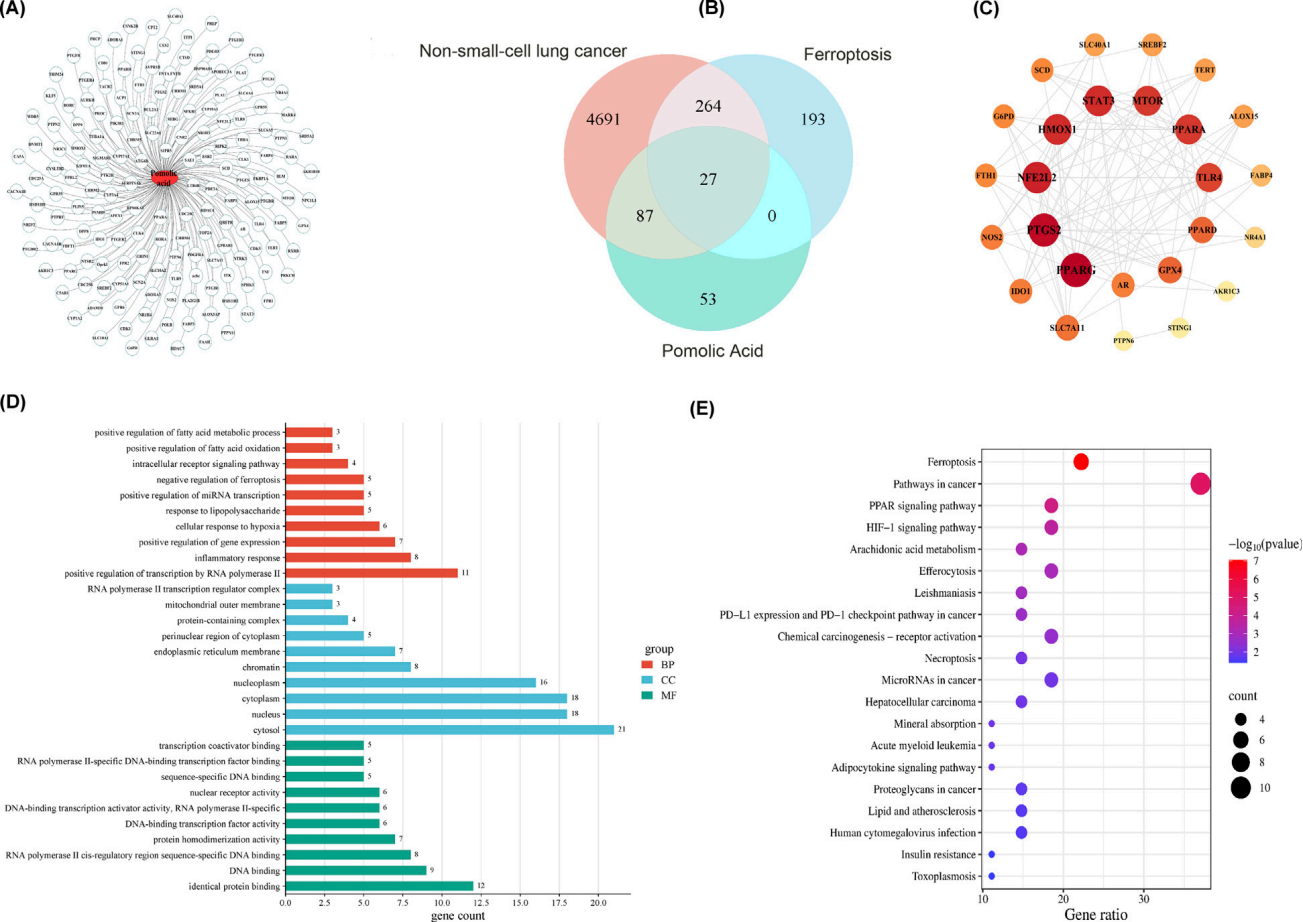
Network pharmacology analysis of PA chemical components for the treatment of non-small cell lung cancer. **(A)** PA component-target diagram. **(B)** Venn diagram of compound targets of PA disease targets. **(C)** PPI network of disease-drug targets. **(D)** GO enrichment analysis of key targets (list the top 10). **(E)** KEGG pathway enrichment analysis of key targets (list the top 20 pathways).

Comprehensive network pharmacology analyses are conducted to explore the molecular mechanisms by which PA acts on NSCLC. Initially, querying the SwissTargetPrediction, SuperPred, and TargetNet databases yields 167 potential protein targets of PA. The predicted interactions among these targets are visualized (Figure 2A). Separately, searches in the OMIM, GeneCards, and DisGeNET databases identify 5,069 genes associated with NSCLC, while the FerrDb database provides a list of 484 human genes related to ferroptosis.

Venn analysis comparing these three gene sets (PA targets, NSCLC-associated genes, ferroptosis-related genes) identifies an intersection of 27 common genes (Figure 2B). A protein-protein interaction (PPI) network is constructed based on these 27 shared genes, revealing a network containing 26 nodes and 107 edges (Figure 2C). Analysis of this network identifies the top 15 genes with the highest node degree centrality, considered core targets or hub genes.

1. Functional enrichment analysis is then performed on these 15 core targets. Gene Ontology (GO) analysis indicates enrichment in various biological processes (Figure 2D). In contrast, the Kyoto Encyclopedia of Genes and Genomes (KEGG) pathway analysis highlights significant enrichment in pathways pertinent to both cancer progression and ferroptosis regulation (Figure 2E). These bioinformatic findings suggest a potential link between PA, ferroptosis, and NSCLC pathophysiology.

### 3.3 Ferroptosis inhibitors attenuate PA-induced cell death *in vitro*

**FIGURE 3:**
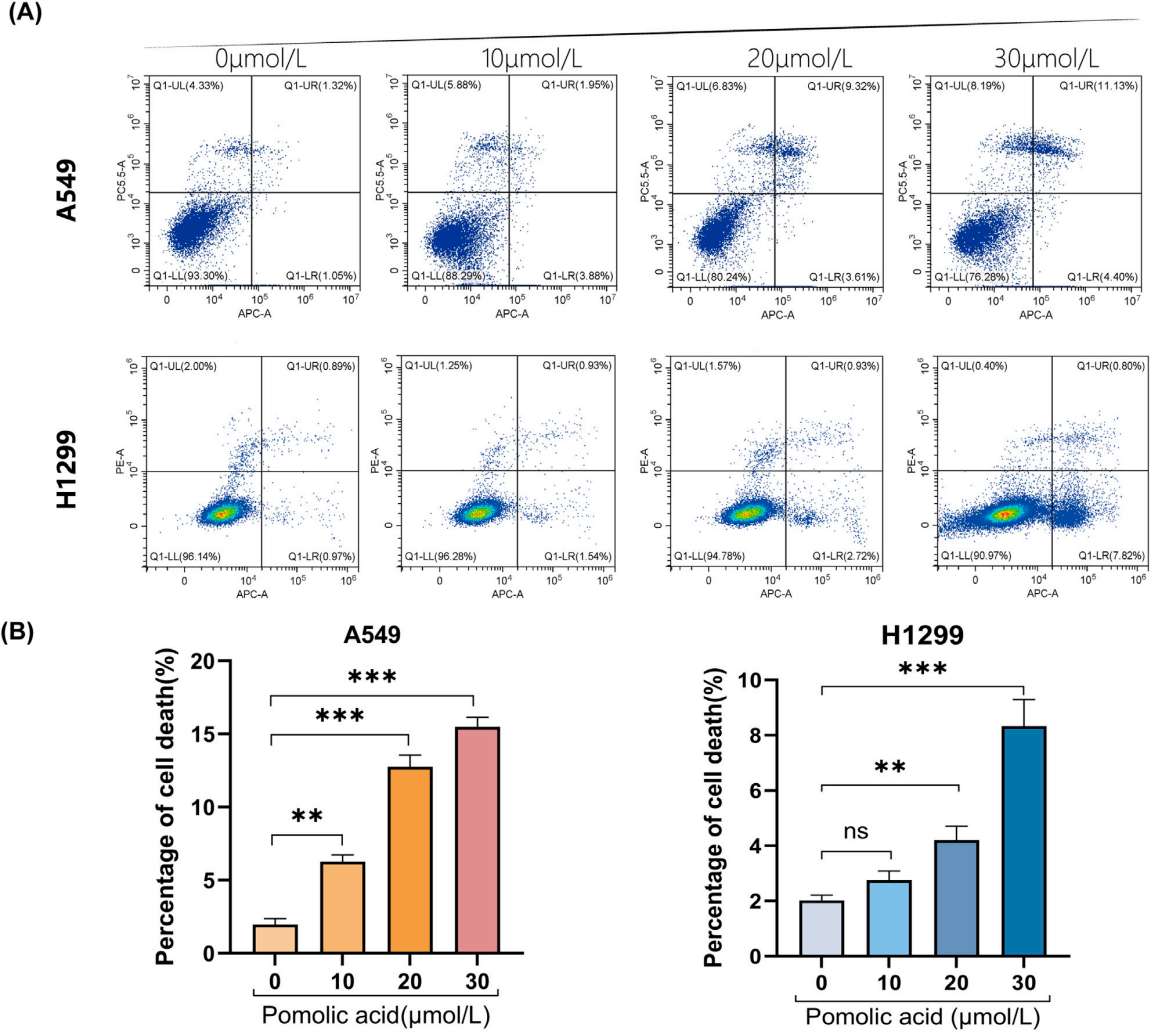
Representative results **(A)** and quantitative analysis **(B)** of Annexin V-APC/PI staining after 24 h of PA treatment. *P < 0.05, **P < 0.01, ***P < 0.001.

To characterize the nature of cell death induced by PA in NSCLC cells, Annexin V-APC/Propidium Iodide (PI) double staining coupled with flow cytometry is employed. This analysis reveals that PA treatment significantly increases the total percentage of non-viable (Annexin V-positive and/or PI-positive) A549 and H1299 cells compared to vehicle-treated controls (Figures 3A,B). Notably, a discrepancy exists between the proportion of cells undergoing classical apoptosis and the overall reduction in cell viability measured by the CCK-8 assay (Figures 1A,B, 3A,B). Given that ferroptosis involves non-apoptotic pathways culminating in compromised cell membrane integrity, it is hypothesized that ferroptosis significantly contributes to PA-induced cytotoxicity and may account for this apparent difference between assays measuring distinct cellular endpoints.

**FIGURE 4:**
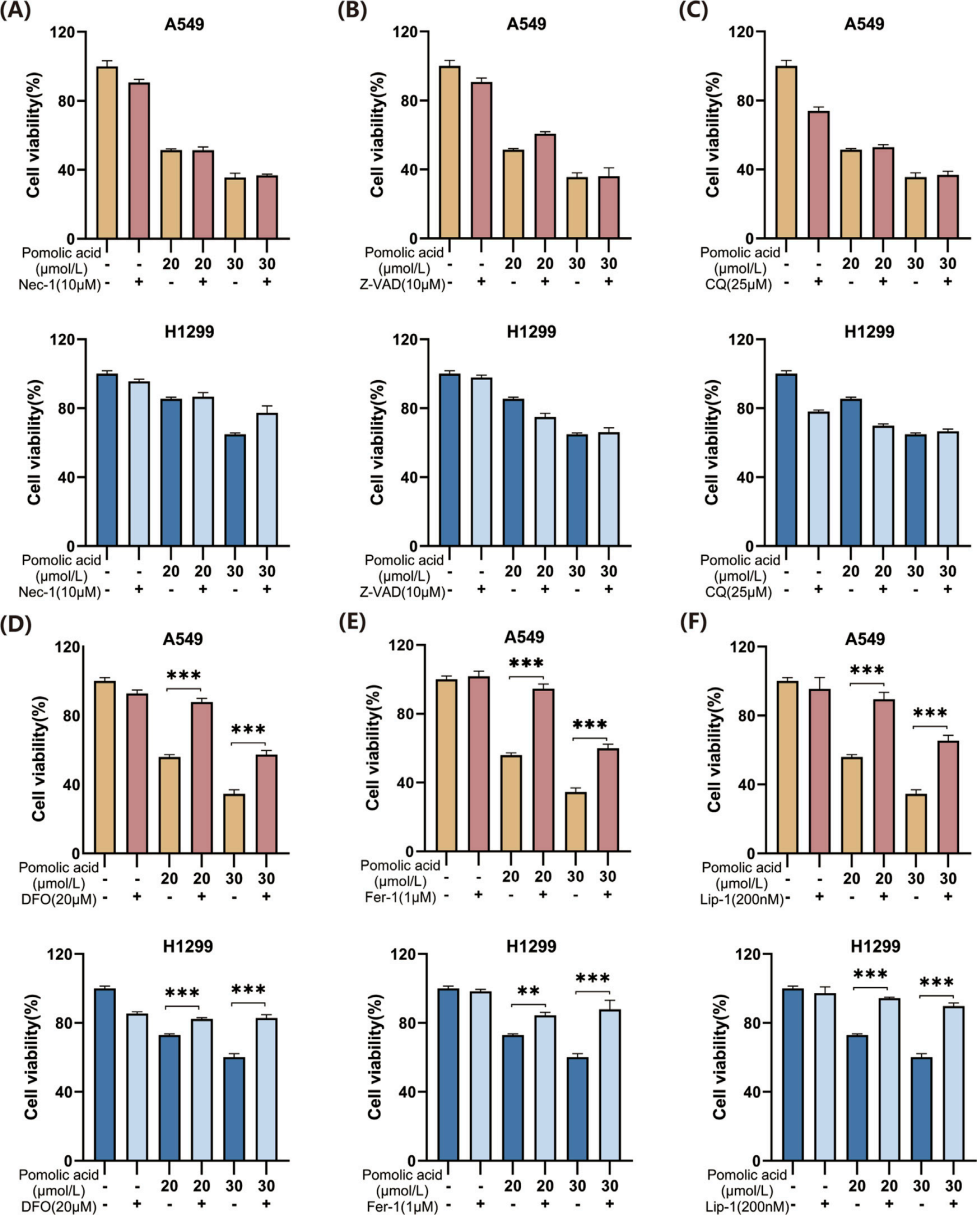
Effects of PA alone or in combination with different cell death inhibitors on lung cancer cell viability. **(A)** A549 and H1299 cells were treated with PA with or without Nec-1 (10 μM) for 24 h, and cell viability was detected. **(B)** A549 and H1299 cells were treated with PA with or without 10 μM Z-VAD for 24 h, and cell viability was detected. **(C)** A549 and H1299 cells were treated with PA with or without CQ (25 μM) for 24 h, and cell viability was detected. **(D)** A549 and H1299 cells were treated with PA with or without DFO (20 μM) for 24 h, and cell viability was analyzed. **(E)** A549 and H1299 cells were treated with PA with or without fer1 (1 μM) for 24 h, and cell viability was analyzed. **(F)** A549 and H1299 cells were treated with PA with or without Lip-1 (200 nM) for 24 h, and cell viability was detected. *P < 0.05, **P < 0.01, ***P < 0.001.

To discern the primary cell death pathway involved, the effect of specific inhibitors targeting different regulated cell death modalities is examined. Cells are co-incubated with PA and inhibitors of necroptosis (necrostatin-1; Nec-1), autophagy (chloroquine; CQ), apoptosis (the pan-caspase inhibitor Z-VAD-FMK; Z-VAD), or ferroptosis (the iron chelator deferoxamine, DFO; or the radical-trapping antioxidants ferrostatin-1, Fer-1, and liproxstatin-1, Lip-1). The viability assays demonstrate that inhibition of necroptosis (Nec-1), apoptosis (Z-VAD), or autophagy (CQ) does not significantly rescue A549 or H1299 cells from PA-induced death (Figures 4A–C). However, co-treatment with any of the ferroptosis inhibitors (DFO, Fer-1, or Lip-1) markedly attenuates PA-induced cell death in both NSCLC cell lines (Figures 4D–F). These findings strongly suggest that ferroptosis is a principal mechanism underlying PA-mediated cytotoxicity in NSCLC cells.

### 3.4 PA treatment alters ferroptosis-related markers in NSCLC cells

**FIGURE 5:**
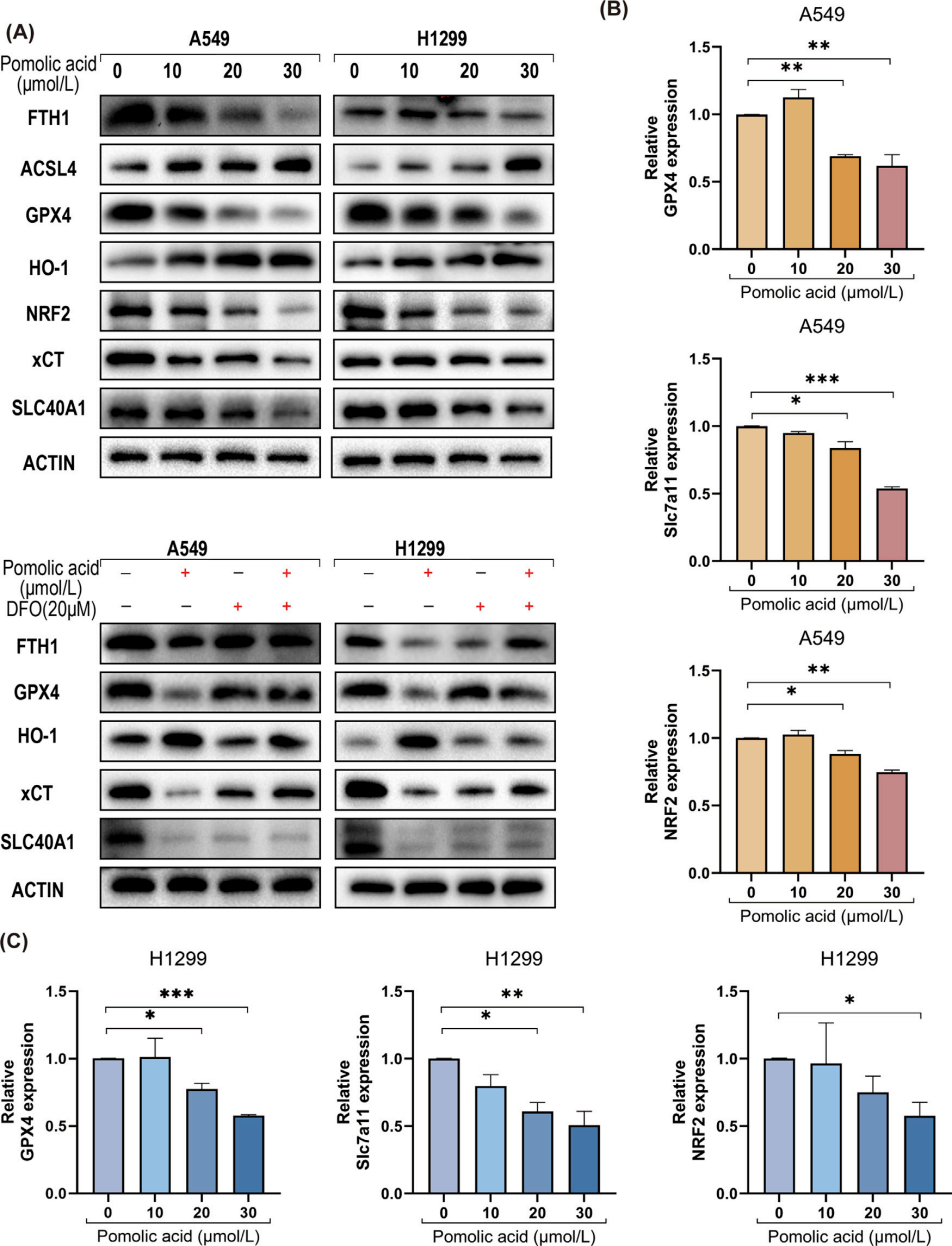
PA induces ferroptosis in lung cancer cells. **(A)** Western blotting was used to detect the expression of ferroptosis-related proteins in lung cancer cells after 24 h of PA with or without DFO (20 μM). **(B)** A549 cells were treated with varying concentrations of PA for 24 h, and qRT-PCR was conducted to assess the mRNA expression of ferroptosis-related genes NRF2, SLC7A11, and GPX4. **(C)** H1299 cells were treated with varying concentrations of PA for 24 h, and qRT-PCR was conducted to assess the mRNA expression of ferroptosis-related genes NRF2, SLC7A11, and GPX4. *P < 0.05, **P < 0.01, ***P < 0.001.

Based on the finding that ferroptosis inhibitors rescue NSCLC cells from PA-induced death (Figures 4D–F), the effects of PA on key molecular regulators and markers of ferroptosis are investigated. Western blot analysis reveals that PA treatment significantly decreases the protein expression levels of several critical anti-ferroptosis factors: glutathione peroxidase 4 (GPX4), ferroportin (SLC40A1), the cystine/glutamate antiporter subunit xCT (SLC7A11), ferritin heavy chain 1 (FTH1), and nuclear factor E2-related factor 2 (NRF2). Concurrently, PA treatment leads to a significant increase in the protein levels of pro-ferroptosis factors heme oxygenase 1 (HO-1) and acyl-CoA synthetase long-chain family member 4 (ACSL4) in both A549 and H1299 cells (Figure 5A). Importantly, co-treatment with the iron chelator deferoxamine (DFO) partially counteracts these PA-induced alterations in protein expression (Figure 5A).

Consistent with the protein level changes, quantitative real-time PCR (qPCR) analysis shows that PA significantly downregulates the mRNA expression levels of the crucial anti-ferroptosis genes GPX4, SLC7A11, and NRF2 in both NSCLC cell lines in a dose-dependent manner (Figures 5B,C).

**FIGURE 6:**
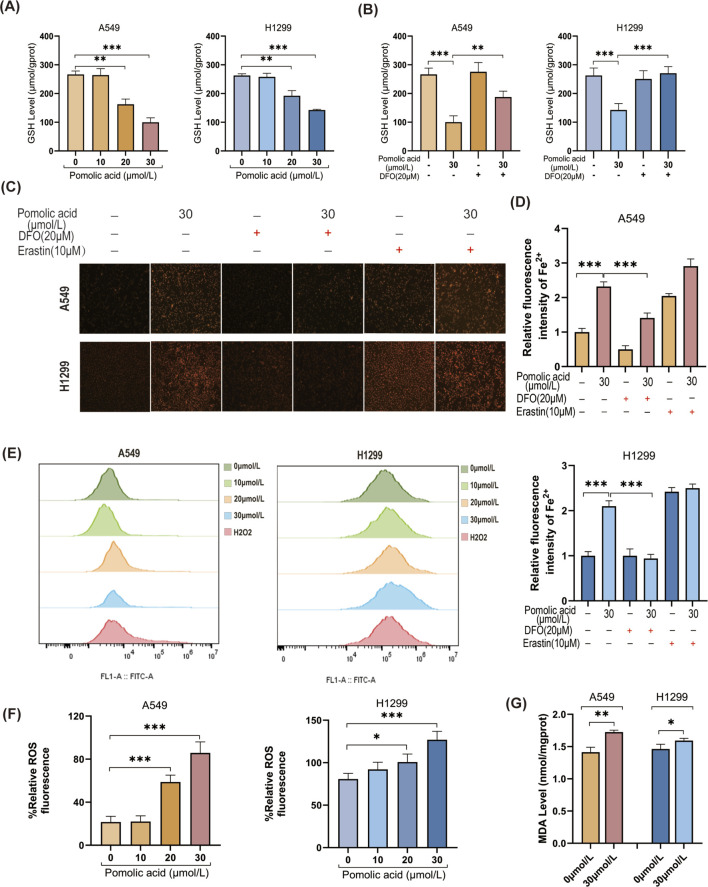
Ferroptosis contributes to PA-induced lung cancer cell death. **(A)** GSH levels were detected after A549 and H1299 cells were treated with PA for 24 h. **(B)** A549 and H1299 cells were treated with PA with or without DFO (20 μM) for 24 h, and GSH levels were analyzed. **(C)** PA with or without DFO (20 μM) and Erastin 10 (10 μM) was used to treat A549 and H1299 cells for 24 h, and the intracellular Fe2+ level was analyzed (×200). **(D)** Quantitative statistical analysis of Fe2+ fluorescence. **(E)** A549 and H1299 cells were treated with PA for 24 h, and ROS levels were detected by flow cytometry using 100 μM H_2_O_2_ as a positive indicator. **(F)** Quantitative analysis of ROS levels was performed. **(G)** MDA levels were measured after 24 h of PA (30 μm/L) treatment. *P < 0.05, **P < 0.01, ***P < 0.001.

Key biochemical hallmarks are assessed to confirm the further induction of ferroptosis. PA treatment leads to a significant depletion of intracellular reduced GSH levels (Figure 6A), and this depletion is mitigated by co-treatment with DFO (Figure 6B). Direct visualization of intracellular labile ferrous iron (Fe2+) using the FerroOrange probe demonstrates a significant increase in Fe2+ levels upon PA treatment, which is effectively reversed by DFO (Figures 6C,D). In addition, PA treatment significantly increases intracellular ROS levels and leads to a greater accumulation of the lipid peroxidation product MDA (Figures 6E–G). Collectively, these data indicate that PA treatment triggers multiple characteristic features of ferroptosis in NSCLC cells.

## 4 Discussion

Cancer continues to pose a significant challenge to human health, with NSCLC standing as a primary contributor to global cancer-related mortality. Despite the availability of multiple treatment modalities, existing therapies exhibit substantial limitations, and the 5-year survival rate for patients with NSCLC remains disappointingly low (Wang et al., 2010). This underscores the urgent imperative for developing novel pharmacological agents and therapeutic strategies to combat this disease more effectively.

Natural products represent a valuable and increasingly explored resource for anti-cancer drug discovery, often exhibiting favorable safety and efficacy profiles. Indeed, natural products form a cornerstone of contemporary cancer treatment, with a substantial number of clinically approved anti-cancer drugs deriving from plants, animals, microorganisms, or marine organisms (Nobili et al., 2009). Prominent examples include plant-derived agents like vincristine, irinotecan, etoposide, and paclitaxel (Kintzios, 2007), as well as microbial products such as actinomycin D, mitomycin C, bleomycin, doxorubicin, and L-asparaginase (Giurini et al., 2024). Cytarabine, derived from a marine sponge, is a key contribution to marine natural products (Bergmann and Feeney, 2002). Ongoing research and development efforts focusing on next-generation taxanes, anthracyclines, vinca alkaloids, camptothecins, and epothilones continuously enrich the therapeutic arsenal based on natural scaffolds. Some of these agents achieve clinical application, while others undergo evaluation in clinical trials.

Certain classes of natural products, such as pentacyclic triterpenoids, which include PA, possess a broad spectrum of biological activities, encompassing antioxidant, antimicrobial, anti-inflammatory, and notable anti-cancer potential, even though they may occur in low abundance naturally. However, compounds like PA often face challenges related to poor aqueous solubility and low bioavailability, which hinder their clinical translation (Li et al., 2023; Chang et al., 2024a). To surmount these obstacles, nanotechnology-based delivery systems are actively investigated as a means to enhance their therapeutic performance (Soica et al., 2014; Valdés et al., 2016). Diverse nanocarrier strategies, encompassing cyclodextrin complexation, micro/nano-emulsions, liposomal encapsulation, and polymeric nanoparticles, show promise for improving the pharmacokinetic properties of these molecules (Kaps et al., 2021; Zhang et al., 2023). Cutting-edge research also explores sophisticated systems like liposome-triterpene or nanolipid-triterpene conjugates linked to microbubbles, enabling targeted delivery via techniques like sonoporation, which could potentially heighten therapeutic effectiveness (Soica et al., 2014). Such nanocarrier platforms generally aim to increase solubility, enhance bioavailability, improve stability, and simultaneously reduce systemic toxicity and adverse effects (Valdés et al., 2016; Wang et al., 2024a). The continued advancement of these innovative nanotechnological approaches is anticipated to unlock the full therapeutic potential of pentacyclic triterpenes in cancer treatment. Therefore, the investigation of natural products, particularly when synergized with innovative delivery materials, remains a critical avenue for identifying novel, potent, and less toxic anti-cancer therapies.

Focusing specifically on PA, previous literature documents its anti-cancer efficacy in various models. For instance, PA induces apoptosis and curtails migration in glioblastoma cells (Guimaraes et al., 2017) and also inhibits proliferation while promoting apoptosis in breast cancer cells (Youn et al., 2012). Furthermore, compelling evidence indicates that PA retains significant anti-cancer activity even against drug-resistant cancer cells, such as doxorubicin-resistant prostate cancer lines (Nobili et al., 2009). In the present study, PA demonstrably inhibits the viability of NSCLC cells *in vitro*. Moreover, this cytotoxic effect occurs in a concentration-dependent fashion. These observations align with previously published findings, lending further support to the established anti-cancer properties of PA (Nobili et al., 2009; Wang et al., 2010; Kanehisa et al., 2023). The results herein not only expand the documented spectrum of PA’s anti-cancer activity to include NSCLC but also position PA as a candidate for further investigation as a potential novel therapeutic agent for patients with this challenging malignancy.

Investigations into the anti-cancer mechanisms of PA reveal diverse activities across different cancer types. Previous studies report that PA induces apoptosis and reduces migration in glioblastoma cells, potentially through inhibition of the multidrug resistance protein MRP1 (Guimaraes et al., 2017). In breast cancer models, PA reportedly inhibits proliferation and promotes apoptosis via activation of the AMPK, caspase, and PARP pathways (Youn et al., 2012). Other work indicates that PA suppresses breast cancer cell invasion by downregulating the expression of CXC chemokine receptor 4 (CXCR4) (Kim et al., 2017). Additionally, PA exerts anti-cancer effects by interfering with key signaling cascades; for instance, it blocks the NF-κB/ERK/mTOR pathway and inhibits MMP-9 and FAK expression in growth factor-stimulated breast cancer cells (Park et al., 2016). Complementing these findings, the present study demonstrates that PA inhibits colony formation and induces S-phase cell cycle arrest in NSCLC cells. Intriguingly, our flow cytometry data indicate that while PA treatment leads to high levels of overall cell death, the proportion of cells undergoing classical apoptosis appears relatively low. Given that prior research links PA-induced apoptosis in glioma cells to ROS generation (Guimaraes et al., 2017) and recognizing that ferroptosis is a distinct ROS-associated cell death modality (Dixon et al., 2012), we postulate that ferroptosis plays a significant role in PA-induced cytotoxicity in NSCLC cells.

Ferroptosis represents a unique form of iron-dependent regulated cell death, biochemically characterized by depletion of GSH, functional inactivation of the key antioxidant enzyme GPX4, and consequent accumulation of toxic lipid peroxides (Dixon et al., 2012; Wang et al., 2024b). Mitochondria are central players in ferroptosis, contributing through processes such as the tricarboxylic acid (TCA) cycle, oxidative phosphorylation, and iron metabolism regulation (Chang et al., 2023a; Sun et al., 2023). Conditions of iron overload exacerbate mitochondrial ROS production and compromise mitochondrial membrane potential, thereby promoting ferroptotic cell death (Wang et al., 2024b). The NRF2-ARE antioxidant pathway is also critically involved; specifically, iron overload can suppress the binding of the transcription factor NRF2 to antioxidant response elements (AREs) located in the promoter regions of essential anti-ferroptosis genes like GPX4 and SLC7A11 (Pang et al., 2024; Wang et al., 2024b). Key molecular regulators of ferroptosis include the GPX4 mentioned above, which acts as a primary suppressor (Yang et al., 2014; Seibt et al., 2019), alongside transcription factors NRF2 (generally protective) and BACH1 (generally pro-ferroptotic). BACH1, for example, promotes ferroptosis by modulating the expression of genes involved in iron homeostasis, the GSH-GPX4 pathway, and the ferroptosis suppressor protein 1 (FSP1)-Coenzyme Q10 (CoQ) pathway (Nishizawa et al., 2023). Additional layers of regulation involve pathways governing iron metabolism, GSH biosynthesis, lipid metabolism, and mitochondrial integrity (Chen et al., 2021; Chang et al., 2023b). Fundamentally, ferroptosis is driven by overwhelming lipid peroxidation, typically initiated by the confluence of excess ROS and labile iron accumulation. Therefore, molecules influencing iron or ROS metabolism are pivotal regulators of this process. Both elevated ROS and iron accumulation are considered hallmarks of ferroptosis, and maintaining ROS balance is crucial for cellular homeostasis and fate decisions (Chen et al., 2021). The unique biochemical features of ferroptosis make it an attractive therapeutic target in cancer, as malignant cells often display heightened basal levels of iron and ROS, potentially sensitizing them to ferroptosis-inducing agents (Battaglia et al., 2020).

Our initial bioinformatics analysis suggests a mechanistic link between PA, NSCLC, and ferroptosis (Li et al., 2024). Specifically, KEGG pathway analysis reveals significant enrichment of PA’s potential targets in pathways related to both ferroptosis and cancer processes. This bioinformatic insight prompts the hypothesis that PA exerts its anti-NSCLC effects, at least in part, by modulating ferroptosis. Consequently, we experimentally investigate the expression of key ferroptosis-related genes and the characteristic biochemical hallmarks of ferroptosis in NSCLC cells following PA treatment.

Ferroptosis is mechanistically defined as an iron-dependent, non-apoptotic form of regulated cell death characterized by distinct morphological and molecular features. A central gatekeeper against ferroptosis is glutathione peroxidase 4 (GPX4), an enzyme critical for tumor cell survival that utilizes reduced GSH to detoxify lipid peroxides. When GPX4 activity is compromised, toxic lipid hydroperoxides accumulate, leading to ferroptotic cell death. This process can typically be prevented or reversed by iron chelators, such as deferoxamine (DFO), or lipid-soluble antioxidants like vitamin E (Yang et al., 2014). Another crucial anti-ferroptosis component is the cystine/glutamate antiporter, system xc-, whose functional subunit is SLC7A11 (also known as xCT). System xc-imports extracellular cystine, which is intracellularly reduced to cysteine, the rate-limiting substrate for the synthesis of GSH. Therefore, SLC7A11 activity is vital for maintaining GSH levels and GPX4 function (Liu et al., 2021). Consistent with its protective role, ferroptosis can be triggered in drug-resistant cancer cells (e.g., cisplatin-resistant HNC cells) by inhibiting or knocking down SLC7A11 (Wang et al., 2023). The system xc-/GSH/GPX4 axis represents a core antioxidant defense pathway protecting against ferroptosis, and its upregulation often supports tumor cell survival and proliferation. Conversely, inhibiting this axis sensitizes cells to ferroptosis. Elevated expression or activity of GPX4 and SLC7A11 is frequently observed in tumors and correlates with resistance to ferroptosis induction (Wen et al., 2024).

In the current study, treatment of H1299 and A549 NSCLC cells with PA leads to a reduction in both the protein and mRNA expression levels of GPX4 and SLC7A11. This finding is entirely consistent with the induction of ferroptosis, as it signifies the suppression of key cellular defenses against this process. We provide evidence, possibly for the first time in the context of PA and NSCLC, that PA’s anti-cancer activity involves ferroptosis induction. This conclusion is supported by multiple lines of experimental data: PA treatment triggers significant ROS generation, depletes intracellular GSH, increases lipid peroxidation (indicated by elevated MDA levels), and causes accumulation of intracellular labile ferrous iron (Fe2+). Furthermore, these biochemical changes are accompanied by the downregulation of critical negative regulators of ferroptosis, namely, GPX4 and the transcription factor NRF2 (which controls the expression of multiple antioxidant genes, including GPX4 and SLC7A11). The observed increase in ROS production also resonates with previous studies linking PA’s effects to ROS generation (Guimaraes et al., 2017). Collectively, our findings strongly suggest that ferroptosis is a principal mechanism contributing to PA-induced cell death in NSCLC cells.

## 5 Conclusion

In conclusion, the findings presented in this study demonstrate that PA exhibits potent, concentration-dependent anti-tumor activity against NSCLC cells *in vitro*. Our results strongly indicate that this efficacy is primarily mediated through the robust induction of ferroptosis. Mechanistically, PA treatment leads to the downregulation of critical negative regulators of ferroptosis, including GPX4 and SLC7A11. This suppression results in the depletion of intracellular GSH and a compromised ability to neutralize toxic lipid peroxides, ultimately triggering ferroptosis cell death. Concurrently, PA also induces cell cycle arrest, which likely enhances its overall anti-proliferative effect and highlights a potential dual mechanism involving both ferroptosis induction and proliferation inhibition. These collective findings underscore the therapeutic promise of PA as a novel anti-cancer agent derived from a natural source. This research provides a solid foundation for continued preclinical investigation of PA, aiming ultimately to translate its ferroptosis-mediated anti-cancer properties into effective *in vivo* therapeutic strategies for lung cancer.

It is important to acknowledge certain limitations of the current study. First, while our *in vitro* data confirm PA’s capacity to induce ferroptosis in NSCLC cells, our mechanistic investigation primarily focuses on the canonical GPX4-GSH axis. We do not exclude the potential contribution of non-canonical ferroptosis regulatory pathways, such as the FSP1-CoQ10-NAD(P)H system, the GCH1-BH4-DHFR axis, mitochondrial DHODH activity, non-classical roles of p53, or alterations in autophagy-mediated iron and lipid handling. Future research endeavors should aim to delineate PA’s precise molecular targets within these broader networks, possibly utilizing unbiased approaches like affinity-based proteomics or genome-wide CRISPR/Cas9 screens to identify direct interactors and further elucidate PA’s mechanism of action. Second, although we observe concomitant ferroptosis induction, cell cycle arrest, and proliferation inhibition, the intricate interplay and potential crosstalk among these processes, as well as other regulated cell death pathways (e.g., apoptosis, necroptosis) following PA treatment, require further investigation. Subsequent studies employing combinatorial genetic and pharmacological approaches to inhibit specific pathways will be valuable for dissecting the relative contribution of ferroptosis versus other mechanisms to PA’s overall effect. Third, the present findings are based entirely on *in vitro* cell culture models. Crucial parameters such as the pharmacokinetics, biodistribution, and *in vivo* efficacy of PA in inducing ferroptosis within a complex tumor microenvironment remain to be assessed. Therefore, future work necessitates the use of appropriate animal models, including tumor xenografts and potentially genetically engineered mouse models, to evaluate PA’s ability to trigger ferroptosis *in vivo* (assessed via relevant biomarkers like tumor iron levels, lipid ROS, and marker protein expression) and to ascertain its safety profile regarding potential off-target effects in normal tissues.

## Data Availability

The raw data supporting the conclusions of this article will be made available by the authors, without undue reservation.
